# Data for ultimate bearing capacity of concrete-filled steel tubular members and arches by the elastic modulus reduction method

**DOI:** 10.1016/j.dib.2020.105994

**Published:** 2020-07-06

**Authors:** LuFeng Yang, Weiwei Xie, YuFeng Zhao, Jian Zheng

**Affiliations:** a Key Laboratory of Disaster Prevention and Structural Safety of China Ministry of Education, School of Civil Engineering and Architecture, Guangxi University, Nanning, 530004, China; b Guangxi Road and Bridge Engineering Group Co. Ltd, Nanning, 530011, China

**Keywords:** Concrete-filled steel tubular, Circular hollow section, Ultimate bearing capacity, Member, Arch

## Abstract

Homogeneous generalized yield function is adopted in this article to calculate the ultimate bearing capacity of 93 concrete-filled steel tubular components with detailed test data, and the ratios of the ultimate bearing capacity calculated to the tested are presented. Moreover, the incremental nonlinear finite element method and elastic modulus reduction method are adopted to evaluate the ultimate bearing capacity of 11 concrete-filled steel tubular arches, 7 among which with detailed test data. The component data cover those under different loading conditions, material strength and geometric parameters, and the arch data include those under different loading conditions and rise to span ratios. The data provided are useful to investigate the strength of CFST members and arches and to demonstrate the validation of other numerical methods. The current data are considered as a complementary for the main work “Linear elastic iteration technique for ultimate bearing capacity of circular CFST arches” [1].

**Specifications Table**

**Table:** 

**Subject**	Civil and Structural Engineering
**Specific subject area**	Concrete-filled steel tubular (CFST) structures
**Type of data**	Table
**How data were acquired**	Formula calculation, finite element analysis and test data collected
**Data format**	Raw
	Analyzed
**Parameters for data collection**	Data of ultimate bearing capacity of circular CFST members and arches
**Description of data collection**	The ultimate bearing capacity of circular CFST members evaluated by homogeneous generalized yield function under different loadings and geometric parameters;
	The ultimate bearing capacity of CFST arches estimated by the incremental nonlinear finite element method and elastic modulus reduction method under different loading conditions and rise to span ratios.
**Data source location**	Nanning, China
	Rajasthan, India
	Beijing, China
	New South Wales, Australia
	Harbin, China
	Fuzhou, China
**Data accessibility**	With the article
**Related research article**	L.F. Yang, W.W. Xie, Y.F. Zhao, J. Zheng. Linear elastic iteration technique for ultimate bearing capacity of circular CFST arches. https://doi.org/10.1016/j.jcsr.2020.106135. [1]

**Value of the Data**

• The data provided are useful to investigate the ultimate bearing capacity and generalized yield function (GYF) of concrete-filled steel tubular (CFST) members under different loading conditions
• The data presented are valuable to investigate the ultimate bearing capacity of CFST arches under different loading conditions
• The data may be useful to researchers interested in CFST structures.
• The data provided can serve as benchmark to validate other numerical methods for CFST structures.

## 1 Data

### 1.1 Data of ultimate bearing capacity of CFST members

The ultimate bearing capacities of 72 CFST members under axial loads, 12 CFST members under pure bending and 9 CFST members under eccentric compression with detailed test data in Lit. [2], [3], [4] are calculated by the homogeneous GYF${\bar{f}}_{4}\left(n_{x},m_{y}\right)$:

(1) $${\bar{f}}_{4}\left(n_{x},m_{y}\right)=1.0066n_{x}^{4}+3.4516n_{x}^{3}m_{y}+6.2752n_{x}^{2}m_{y}^{2}+2.2168m_{y}^{3}n_{x}+1.0088m_{y}^{4}$$

(2) $$n_{x}=\frac{N_{x}}{N_{\text{px}}},\;\;m_{y}=\frac{M_{y}}{M_{p}}$$

where *n*_x_ and *m*_y_ are normalized internal forces. *N*_x_ and *M*_y_ are the axial force and bending moment, respectively. *N*_px_ and *M*_p_ represent the full plastic axial force and bending moments, respectively.

The ratios of the calculated to the tested are obtained under axial loading, pure bending and eccentric compression. Then, the calculated data and the test data for CFST members under axial loading, pure bending and eccentric compression are listed in Table 1-3, respectively. The *D, t, l* and *f*_y_ in Table 1-3 are the diameter, wall thickness, calculation length and yield strength of steel. $f_{c}^{\prime }$, *f*_cu,k_ and *e* in Table 1-3 are the concrete cylinder strength, concrete cube strength and eccentricity.

**Table 1:** Ultimate loading capacity of CFST members under axial compression (kN).

*D* (mm)	*t* (mm)	*l* (mm)	*f*_y_ (MPa)	$f_{c}^{\prime }$ (MPa)	Test in Lit. [2]	HGYF
47.28	1.87	340	360	25.15	215	158.69
47.28	1.87	340	360	28.89	215	164.08
47.28	1.87	340	360	28.22	210	163.09
47.28	1.87	340	360	27.15	167	161.53
47.28	1.87	340	360	25.33	178	158.94
47.28	1.87	340	360	22.22	187	154.79
47.28	1.87	340	360	29.02	145	164.27
47.28	1.87	340	360	28.22	166	163.09
47.28	1.87	340	360	29.73	176	165.34
47.28	1.87	340	360	28.53	171	163.55
47.28	1.87	340	360	25.2	168	158.76
47.28	1.87	340	360	22.44	160	155.07
89.32	2.74	340	360	25.15	610	484.79
89.32	2.74	340	360	28.89	635	506.13
89.32	2.74	340	360	28.22	630	502.25
89.32	2.74	340	360	27.15	524	496.11
89.32	2.74	340	360	25.33	494	485.80
89.32	2.74	340	360	22.22	530	468.73
89.32	2.74	340	360	29.02	540	506.88
89.32	2.74	340	360	28.22	494	502.25
89.32	2.74	340	360	29.73	560	511.02
89.32	2.74	340	360	28.5	571	503.87
89.32	2.74	340	360	25.2	582	485.07
89.32	2.74	340	360	22.44	557	469.91
112.56	2.89	340	360	25.15	754	692.27
112.56	2.89	340	360	28.89	730	727.57
112.56	2.89	340	360	28.22	745	721.19
112.56	2.89	340	360	27.15	635	711.04
112.56	2.89	340	360	25.33	720	693.95
112.56	2.89	340	360	22.22	650	665.32
112.56	2.89	340	360	29.02	686	728.81
112.56	2.89	340	360	28.22	716	721.19
112.56	2.89	340	360	29.73	681	735.60
112.56	2.89	340	360	28.53	687	724.14
112.56	2.89	340	360	25.2	700	692.73
112.56	2.89	340	360	22.44	674	667.31
47.28	1.87	340	360	37.6	250	177.68
47.28	1.87	340	360	40	225	181.59
47.28	1.87	340	360	37.77	246	177.96
47.28	1.87	340	360	35.68	177	174.60
47.28	1.87	340	360	36.67	192	176.18
47.28	1.87	340	360	38.31	165	178.83
47.28	1.87	340	360	31.42	157	167.91
47.28	1.87	340	360	38.23	156	178.70
47.28	1.87	340	360	36.88	162	176.52
47.28	1.87	340	360	30.88	190	167.08
47.28	1.87	340	360	32.44	203	169.49
47.28	1.87	340	360	34.66	194	172.97
89.32	2.74	340	360	37.6	644	557.96
89.32	2.74	340	360	40	620	572.57
89.32	2.74	340	360	37.77	650	558.99
89.32	2.74	340	360	35.68	599	546.35
89.32	2.74	340	360	36.67	620	552.32
89.32	2.74	340	360	38.31	605	562.27
89.32	2.74	340	360	31.42	603	520.94
89.32	2.74	340	360	38.23	577	561.78
89.32	2.74	340	360	36.88	552	553.59
89.32	2.74	340	360	30.88	613	517.76
89.32	2.74	340	360	32.44	599	526.97
89.32	2.74	340	360	34.66	605	540.22
112.56	2.89	340	360	37.6	822	812.07
112.56	2.89	340	360	40	788	835.71
112.56	2.89	340	360	37.77	801	813.74
112.56	2.89	340	360	35.68	785	793.25
112.56	2.89	340	360	36.67	755	802.94
112.56	2.89	340	360	38.31	757	819.05
112.56	2.89	340	360	31.42	735	751.85
112.56	2.89	340	360	38.23	727	818.26
112.56	2.89	340	360	36.88	747	805.00
112.56	2.89	340	360	30.88	745	746.64
112.56	2.89	340	360	32.44	758	761.71
112.56	2.89	340	360	34.66	770	783.29

**Table 2:** Ultimate loading capacity of CFST members under pure bending (kN•m).

*D* (mm)	*t* (mm)	*l* (mm)	*f*_y_ (MPa)	*f*_cu,k_ (MPa)	Test in Lit. [3]	HGYF
140	3	840	235	51.5	19.8	21.17
140	3	840	235	51.5	21.6	21.17
140	3	1680	235	51.5	21.5	21.17
140	3	840	235	51.5	22.1	21.17
140	3	1680	235	51.5	20.7	21.17
140	3	1680	235	51.5	20.4	21.17
180	3	900	235	62.6	33.9	39.88
180	3	900	235	62.6	34.9	39.88
180	3	1800	235	62.6	32.2	39.88
180	3	900	235	62.6	40.6	39.88
180	3	1800	235	62.6	36.2	39.88
180	3	1800	235	62.6	36.3	39.88

**Table 3:** Ultimate loading capacity of CFST members under eccentric compression (kN).

*D* (mm)	*t* (mm)	*l* (mm)	*e* (mm)	*f*_y_ (MPa)	$f_{c}^{\prime }$ (MPa)	Test in Lit. [4]	HGYF
240	6	720	120.0	489	31.5	1277	1331.74
360	6	1080	60.1	498	31.5	4294	4228.85
480	6	1440	240.0	468	31.5	3323	3066.18
600	6	1800	300.0	517	31.5	4590	4434.22
240	6	720	120.0	489	59	1438	1517.41
360	6	1080	180.0	498	59	2537	2638.99
480	6	1440	240.0	468	59	3895	3719.44
300	12	900	150.0	479	31.5	3683	2816.71
480	12	1440	240.0	489	31.5	5135	5326.95

The mean and c.o.v. of the aforementioned ratios are 0.96 and 0.11, respectively.

### 1.2 Data of ultimate bearing capacity of CFST arches under in-plane loads

The CFST arches are under in-plane loading with different rise to span ratios. The arch axis equation is of parabolic curves with span length of *L* = 9 m. The outer diameter and wall thickness of the steel tube are 159 mm and 4.5 mm, respectively, and its yield strength *f*_y_ and elastic modulus *E*_s_ are 376.2 MPa and 204 × 10^3^MPa, respectively. The cubic compression strength *f*_cu_ and elastic modulus *E*_c_ of the core concrete are 41.6 MPa and 31.3 × 10^3^MPa, respectively. The ultimate bearing capacity of the CFST arches were obtained by the incremental nonlinear finite element method (INFEM), and the elastic modulus reduction method (EMRM) and test data in Lit. [5], respectively, as illustrated in Table 4.

**Table 4:** Ultimate bearing capacity of the arches with different rise to span ratios.

Loading position	rise to span ratio	Test/kN in Lit. [5]	INFEM /kN	EMRM/kN
1/4 span	1/9	132.5	140.5	130.9
	1/7.5	–	149.1	135.2
	1/6	163.5	158.1	139.7
	1/4.5	165	167.1	144.0
	1/3	–	174.3	148.1
Crown	1/9	103.4	113.1	99.4
	1/7.5	–	116.5	101.0
	1/6	110.7	119.9	103.9
	1/4.5	106.8	123.0	105.0
	1/3	–	124.6	103.7

### 1.3 Data of ultimate bearing capacity of a CFST arch under spatial loads

The CFST arch is under spatial loadings. The in-plane concentrated loadings are applied at five points uniformly distributed along the span while the out-of-plane concentrated loading, vertical to the arch plane and 0.1 times of the in-plane loading, is applied at the crown. The arch axis is of parabolic curve, *y* = 8*×*^2^*/*75 with length of span 7.5 m and the height of the arch 1.5 m. The outer diameter and wall thickness of the steel tube are 121 mm and 4.5 mm, respectively, with the yield strength *f*_y_=322 MPa and the elastic modulus *E*_s_ =2.06 × 10^5^MPa. The cubic compression strength *f*_cu_ and the elastic modulus *E*_c_ of the core concrete are 66.7 MPa and 30.0 × 10^3^MPa, respectively. The ultimate bearing capacity of the arch obtained by the elastic modulus reduction method and the test data in Lit. [6] is 102.4 kN and 97.1 kN,respectively, with relative error of 5.5%.

### 1.4 Data under different material strengths and nominal slenderness ratios

The above CFST arches under in-plane loadings were taken into consideration. The nominal slenderness ratio *λ* is defined as follows:

(3) $$\lambda =4L_{0}/D,\;L_{0}=0.36S_{g}$$

where, *L*_0_ is an equivalent calculation length, *S*_g_ is the axis length of arch and *D* the outer diameter of steel tube.

The ultimate bearing capacity of the CFST arches from the INFEM, the EMRM and test data in Lit. [5] were illustrated in Table 5 under different material strengths and nominal slenderness ratios.

**Table 5:** Ultimate bearing capacity of the arches with different material strengths and nominal slenderness ratios.

Loading position	Nominal slenderness ratio	Test/kN in Lit. [5]	INFEM /kN	EMRM/kN	
				Design material strength	Characteristic material strength
Crown	20	–	414.6	314.2	387.9
	40	–	271.2	219.2	270.8
	60	–	195.6	168.7	208.5
	80	–	147.8	136.1	168.2
	84.12	132.5	140.5	130.9	161.8
	100	–	116.0	113.8	140.6
	120	–	94.0	97.6	120.7
	140	–	77.9	85.4	105.6
	160	–	65.7	75.9	93.8
	180	–	56.4	68.2	84.3
	200	–	48.9	62.0	76.6
1/4 span	20	–	435.2	304.2	375.6
	40	–	248.8	188.5	232.9
	60	–	164.8	134.3	166.0
	80	–	119.7	104.1	128.6
	84.12	103.4	113.1	99.4	122.9
	100	–	92.6	86.4	106.8
	120	–	74.6	72.9	90.1
	140	–	61.9	63.0	77.9
	160	–	52.5	55.5	68.6
	180	–	45.3	49.6	61.3
	200	–	39.6	44.8	55.4

## 2 Experimental design and methods

### 2.1 Homogeneous GYF for CFST members

The homogeneous generalized yield function is adopted to obtain the ultimate bearing capacity of CFST components. The full plastic axial force and bending moments *N*_px_ and *M*_p_ in Eq. (2) are determined on the basis of the material and geometric parameters of the CFST members. Then, substituting *n*_x_ and *m*_y_ into Eq. (1), the ultimate bearing capacity of CFST members can be determined conveniently by solving the *N*_x_ or *M*_y_ from the equation ${\bar{f}}_{4}\left(n_{x},m_{y}\right)=1$ in case of the member under axial loading or pure bending, respectively. If the CFST member is exposed to eccentric compression *N*_x_ with eccentricity *e, M*_y_ is defined as *M*_y_= *N*_x_•*e*. Then substituting *n*_x_ and *m*_y_ into Eq. (1), the ultimate bearing capacity of the member can similarly be evaluated by solving the *N*_x_ from the equation ${\bar{f}}_{4}\left(n_{x},m_{y}\right)=1$.

### 2.2 Elastic modulus reduction method for arches

The incremental nonlinear finite element method and elastic modulus reduction method are used to evaluate the ultimate bearing capacity of CFST arches. As the incremental nonlinear finite element method is commonly used and well-known to researchers, only the main procedures of elastic modulus reduction method are introduced as follows:

**Step 1: Conduct linear elastic finite element analysis of CFST arches**

The linear elastic finite element model is developed to calculate the internal forces for CFST arches.

**Step 2: Identification of highly-stressed elements**

The element bearing ratio $r_{k}^{e}$ of each element in CFST arches is determined by the homogeneous GYF under combined actions of axial force and bending moment:

(4) $$r_{k}^{e}=\sqrt[{4}]{{\bar{f}}_{4}\left(n_{x},m_{y}\right)}$$

Then the reference element bearing ratio reads:

(5) $$r_{k}^{0}=r_{k}^{\max}-d_{k}\left(r_{k}^{\max}-r_{k}^{\min}\right)$$

where $r_{k}^{\max}$ and $r_{k}^{\min}$ are the maximum and minimum element bearing ratios in the *k*^th^ iteration, respectively, and *d_k_*denotes the uniformity of the element bearing ratio.

If$r_{k}^{e}>r_{k}^{0}$, the element *e* is considered as highly-stressed one in *k*^th^ iteration, then its elastic modulus should be reduced.

**Step 3: Elastic modulus reduction**

The Elastic moduli of highly-stressed elements in CFST arches are reduced as:

(6) $$E_{k+1}^{e}=\left\{ \begin{array}{cc} E_{k}^{e}\frac{2{\left(r_{k}^{0}\right)}^{2}}{{\left(r_{k}^{e}\right)}^{2}+{\left(r_{k}^{0}\right)}^{2}} & r_{k}^{e}>r_{k}^{0}\\ E_{k}^{e} & r_{k}^{e}\leq r_{k}^{0} \end{array}\right.$$

where $E_{k}^{e}$ and $E_{k+1}^{e}$ are the elastic moduli of the element *e* in the *k*^th^ and (*k* + 1)^th^ iterations, respectively.

**Step 4: Evaluation of ultimate bearing capacity of CFST arches**

The ultimate bearing capacity $P_{k}^{L}$ for *k*^th^ iterative step is determined according to the maximum element bearing ratio, and reads:

(7) $$P_{k}^{L}=P_{0}/r_{k}^{\max}$$

The above iteration process is repeated until the limit loads between two adjacent iterative steps meet the following convergence criterion:

(8) $$\left|\left(P_{k}^{L}-P_{k-1}^{L}\right)/P_{k-1}^{L}\right|\leq \varepsilon ,\;\;k\geq 2$$

where ɛ is the allowable error.

If the criterion of convergence is satisfied at the *m*^th^ iterative step, the ultimate bearing capacity of the CFST arch writes:

(9) $$P_{L}=P_{m}^{L}$$

## Declaration of Competing Interest

This manuscript is original and not published or being considered for publication elsewhere. It will not be copyrighted, submitted, or published elsewhere while acceptance by Date in Brief is under consideration. All authors have directly participated in the research work of this manuscript.

## Appendix Supplementary materials

Image, application 1
